# Fractional calculus and application of generalized Struve function

**DOI:** 10.1186/s40064-016-2560-3

**Published:** 2016-06-29

**Authors:** Kottakkaran Sooppy Nisar, Dumitru Baleanu, Maysaa’ Mohamed Al Qurashi

**Affiliations:** Department of Mathematics, College of Arts and Science, Prince Sattam Bin Abdulaziz University, 11991 Wadi Aldawser, Saudi Arabia; Department of Mathematics and Computer Sciences, Faculty of Arts and Sciences, Çankaya University, 0630 Ankara, Turkey; Department of Mathematics, King Saud University, 12372 Riyadh, Saudi Arabia

**Keywords:** Fractional calculus, Generalized Struve function, Integral transforms, Fractional kinetic equations, Laplace transforms, Primary 26A33, 44A20; Secondary 33E12, 44A10

## Abstract

A new generalization of Struve function called generalized Galué type Struve function (GTSF) is defined and the integral operators involving Appell’s functions, or Horn’s function in the kernel is applied on it. The obtained results are expressed in terms of the Fox–Wright function. As an application of newly defined generalized GTSF, we aim at presenting solutions of certain general families of fractional kinetic equations associated with the Galué type generalization of Struve function. The generality of the GTSF will help to find several familiar and novel fractional kinetic equations. The obtained results are general in nature and it is useful to investigate many problems in applied mathematical science.

## Background

Fractional calculus has found many demonstrated applications in extensive areas of applied science such as dynamical system in control theory, viscoelasticity, electrochemistry, signal processing and model of neurons in biology (Podlubny 1999; Hilfer 2000; Adjabi et al. 2016; Baleanu et al. 2016; Kilbas et al. 2006; Glöckle and Nonnenmacher 1991; Mathai et al. 2010). Recent studies observed that the solutions of fractional order differential equations could model real-life situations better, particularly in reaction-diffusion type problems. Due to the potential applicability to wide variety of problems, fractional calculus is developed to large area of Mathematics physics and other engineering applications. Several researchers have investigated fractional kinetic equations as its possible applications in diverse physical problems. In this connection, one can refer to the monograph by various works (Saichev and Zaslavsky 1997; Haubold and Mathai 2000; Saxena et al. 2002, 2004, 2006; Saxena and Kalla 2008; Chaurasia and Pandey 2008; Gupta and Sharma 2011; Chouhan and Sarswat 2012; Chouhan et al. 2013; Gupta and Parihar 2014). Recently, many papers investigated the solutions of generalized fractional kinetic equations (GFKE) involving various types of special functions. For instance, the solutions of GFKE involving M-series (Chaurasia and Kumar 2010), generalized Bessel function of the first kind (Kumar et al. 2015), Aleph function (Choi and Kumar 2015) and the generalized Struve function of the first kind (Nisar et al. 2016b). Here, in this paper, we aim at presenting the integral transforms and the solutions of certain general families of fractional kinetic equations associated with newly defined Galué type generalization of Struve function.

Galué (2003) introduced a generalization of the Bessel function of order *p* given by

1 $$\begin{aligned} _{a}J_{p}\left( x\right) :=\sum _{k=0}^{\infty }\frac{\left( -1\right) ^{k}}{ \Gamma \left( ak+p+1\right) k!}\left( \tfrac{x}{2}\right) ^{2k+p}, \quad x\in {\mathbb {R}},a\in {\mathbb {N=}}\left\{ 1,2,3,\ldots \right\} \end{aligned}$$

Baricz (2010) investigated Galué-type generalization of modified Bessel function as:

2 $$\begin{aligned} _{a}I_{p}\left( x\right) :=\sum _{k=0}^{\infty }\frac{1}{\Gamma \left( ak+p+1\right) k!}\left( \tfrac{x}{2}\right) ^{2k+p}, \quad x\in {\mathbb {R}},a\in {\mathbb {N}} \end{aligned}$$

The Struve function of order *p* given by

3 $$\begin{aligned} H_{p}\left( x\right) :=\sum _{k=0}^{\infty }\frac{\left( -1\right) ^{k}}{ \Gamma \left( k+3/2\right) \Gamma \left( k+p+\frac{3}{2}\right) }\left( \tfrac{x}{2}\right) ^{2k+p+1}, \end{aligned}$$

is a particular solution of the non-homogeneous Bessel differential equation

4 $$\begin{aligned} x^{2}y^{^{\prime \prime }}\left( x\right) +xy^{^{\prime }}\left( x\right) +\left( x^{2}-p^{2}\right) y\left( x\right) =\frac{4\left( \frac{x}{2} \right) ^{p+1}}{\sqrt{\pi }\Gamma \left( p+1/2\right) } \end{aligned}$$

where $\Gamma$ is the classical gamma function whose Euler’s integral is given by (see, e.g., Srivastava and Choi 2012, Section 1.1):

5 $$\begin{aligned} \Gamma \left( z\right) =\int _{0}^{\infty }e^{-t}t^{z-1}dt, \quad Re\left( z\right) >0 \end{aligned}$$

The Struve function and its more generalizations are found in many papers (Bhowmick 1962, 1963; Kanth 1981; Singh 1974; Nisar and Atangana 2016; Singh 1985, 1988a, b, 1989). The generalized Struve function given by Bhowmick (1962)

6 $$\begin{aligned} H_{l}^{\lambda }\left( x\right) =\sum _{k=0}^{\infty }\frac{\left( -1\right) ^{k}\left( \frac{t}{2}\right) ^{2k+l+1}}{\Gamma \left( \lambda k+l+\frac{3}{2 }\right) \Gamma \left( k+\frac{3}{2}\right) }, \quad \lambda >0 \end{aligned}$$

and by Kanth (1981)

7 $$\begin{aligned} H_{l}^{\lambda ,\alpha }\left( x\right) =\sum _{k=0}^{\infty }\frac{\left( -1\right) ^{k}\left( \frac{x}{2}\right) ^{2k+l+1}}{\Gamma \left( \lambda k+l+ \frac{3}{2}\right) \Gamma \left( \alpha k+\frac{3}{2}\right) }, \quad \lambda>0, \alpha >0 \end{aligned}$$

Singh (1974) found another generalized form as

8 $$\begin{aligned} H_{l,\xi }^{\lambda }\left( x\right) =\sum _{k=0}^{\infty }\frac{\left( -1\right) ^{k}\left( \frac{x}{2}\right) ^{2k+l+1}}{\Gamma \left( \lambda k+ \frac{l}{\xi }+\frac{3}{2}\right) \Gamma \left( k+\frac{3}{2}\right) },\quad \lambda>0, \xi >0 \end{aligned}$$

The generalized Struve function of four parameters was given by Singh (1985) (also, see Nisar and Atangana 2016) as:

9 $$\begin{aligned} H_{p,\mu }^{\lambda ,\alpha }\left( x\right) :=\sum _{k=0}^{\infty }\frac{ \left( -1\right) ^{k}}{\Gamma \left( \alpha k+\mu \right) \Gamma \left( \lambda k+p+\frac{3}{2}\right) }\left( \tfrac{x}{2}\right) ^{2k+p+1},\quad p,\lambda \in {{\mathbb {C}}} \end{aligned}$$

where $\lambda>0,\alpha >0$ and $\mu$ is an arbitrary parameter. Another generalization of Struve function by Orhan and Yagmur (2014, 2013) is,

10 $$\begin{aligned} {\mathcal {H}}_{p,b,c}\left( z\right) :=\sum _{k=0}^{\infty }\frac{\left( -c\right) ^{k}}{\Gamma \left( k+3/2\right) \Gamma \left( k+p+\frac{b}{2} +1\right) }\left( \tfrac{z}{2}\right) ^{2k+p+1}, \quad p,b,c\in {{\mathbb {C}}} \end{aligned}$$

Motivated from (1), (3) and (10), here we define the following generalized form of Struve function named as generalized Galué type Struve function (GTSF) as:

11 $$\begin{aligned} _{a}{\mathcal {W}}_{p,b,c,\xi }^{\alpha ,\mu }\left( z\right) :=\sum _{k=0}^{\infty }\frac{\left( -c\right) ^{k}}{\Gamma \left( \alpha k+\mu \right) \Gamma \left( ak+\frac{p}{\xi }+\frac{b+2}{2}\right) }\left( \tfrac{z}{2}\right) ^{2k+p+1}, \quad a\in {\mathbb {N}},p,b,c\in {{\mathbb {C}}} \end{aligned}$$

where $\alpha>0,\xi >0$ and $\mu$ is an arbitrary parameter and studied fractional integral representations of generalized GTSF.

The generalized integral transforms defined for $x>0$ and $\lambda ,\sigma ,\vartheta \in {{\mathbb {C}}}$ with $\mathfrak {R}{(\lambda )}>0$ are given in Saigo (1977), (also, see Samko et al. 1987) respectively as

12 $$\begin{aligned} \left( I_{0_{+}}^{\lambda ,\sigma ,\vartheta }f\right) (x)=\frac{x^{-\lambda -\sigma } }{\Gamma (\lambda )}\displaystyle \int _{0}^{x}(x-t)^{\lambda -1}{}_{2}F_{1}\left( \lambda +\sigma ,-\vartheta ;\lambda ;1-\frac{t}{x}\right) f(t)dt \end{aligned}$$

and

13 $$\begin{aligned} \left( I_{-}^{\lambda ,\sigma ,\vartheta }f\right) (x)=\frac{1}{\Gamma (\lambda )} \displaystyle \int _{x}^{\infty }(t-x)^{\lambda -1}t^{-\lambda -\sigma }{}_{2}F_{1}\left( \lambda +\sigma ,-\vartheta ;\lambda ;1-\frac{x}{t}\right) f(t)dt, \end{aligned}$$

where $\Gamma (\lambda )$ is the familiar Gamma function (see, *e.g.*, Srivastava and Choi 2012, Section 1.1) and $_pF_q$ is the generalized hypergeometric series defined by (see, *e.g.*, Rainville 1960, p. 73):

14 $$\begin{aligned} \begin{aligned} _pF_q \left[ \begin{aligned}\alpha _1,\,\ldots ,\,\alpha _p&;\\ \beta _1,\,\ldots ,\,\beta _q&; \end{aligned} \,\, z \right] =&\sum _{n=0}^\infty \, {(\alpha _1)_n \cdots (\alpha _p)_n \over (\beta _1)_n \cdots (\beta _q)_n} {z^n \over n!} \\ =&\,\, _pF_q (\alpha _1,\,\ldots ,\,\alpha _p ;\, \beta _1,\,\ldots ,\,\beta _q;\,z), \end{aligned} \end{aligned}$$

$(\lambda )_n$ being the Pochhammer symbol defined (for $\lambda \in {{\mathbb {C}}}$) by (see Srivastava and Choi 2012, p. 2 and p. 5):

15 $$\begin{aligned} \begin{aligned} (\lambda )_n\!:&=\left\{ \begin{aligned}&1 \qquad \qquad \qquad \qquad \qquad (n=0) \\&\lambda (\lambda +1) \ldots (\lambda +n-1) \quad (n \in {\mathbb {N}}) \end{aligned} \right. \\&= \frac{\Gamma (\lambda +n)}{ \Gamma (\lambda )} \quad (\lambda \in {{\mathbb {C}}} \setminus {\mathbb {Z}}_0^-). \end{aligned} \end{aligned}$$

The results given in Kiryakova (1977), Miller and Ross (1993), Srivastava et al. (2006) can be referred for some basic results on fractional calculus. The Fox–Wright function $\;_p\Psi _q$ defined by (see, for details, Srivastava and Karlsson 1985, p. 21)

16 $$\begin{aligned} \begin{aligned} \; _{p}\Psi _{q}\left[ z\right]&= \;_{p}\Psi _{q}\left[ \begin{array}{rr} \left( a_1, \alpha _1\right) ,\ldots ,\left( a_p,\alpha _p\right) ;\\ \\ \left( b_1, \beta _1\right) ,\ldots ,\left( b_q,\beta _q\right) ;\end{array}\; z\right] =\, _{p}\Psi _{q}\left[ \begin{array}{rr} \left( a_i, \alpha _i\right) _{1,p};\\ \\ \left( b_j, \beta _j\right) _{1,q};\end{array}\; z\right] \\&= \sum _{n=0}^{\infty }\frac{\prod \nolimits _{i=1}^{p}\Gamma \left( a_i+\alpha _i n\right) }{\prod \nolimits _{j=1}^{q}\Gamma \left( b_j+\beta _j n\right) }\;\frac{z^n}{n!}, \end{aligned} \end{aligned}$$

where the coefficients $\alpha _1,\,\ldots , \,\alpha _p,\, \beta _1,\,\ldots ,\,\beta _q \in \mathbb {R}^+$ such that

17 $$\begin{aligned} 1+ \sum _{j=1}^q\,\beta _j - \sum _{i=1}^{p}\, \alpha _i \geqq 0. \end{aligned}$$

For more detailed properties of ${}_{p}\Psi _{q}$ including its asymptotic behavior, one may refer to works (for example Kilbas and Sebastian 2008; Kilbas et al. 2002; Kilbas and Sebastian 2010; Srivastava 2007; Wright 1940a, b).

## Fractional integration of (11)

The following lemmas proved in Kilbas and Sebastian (2008) are needed to prove our main results.

### **Lemma 1**

(Kilbas and Sebastian 2008) *Let*$$\lambda ,\sigma ,\vartheta \in {\mathbb {C}}$$*be*$$\ni$$$$\mathfrak {R}{(\lambda )}>0,\mathfrak {R}{(\rho )}>\max [0, \mathfrak {R}{(\sigma -\vartheta )}].$$*Then*$$\exists$$*the relation*

18 $$\begin{aligned} \left( I_{0_{+}}^{\lambda ,\sigma ,\vartheta }t^{\rho -1}\right) (x)=\dfrac{ \Gamma (\rho )\Gamma (\rho +\vartheta -\sigma )}{\Gamma (\rho -\sigma )\Gamma (\rho +\lambda +\vartheta )}x^{\rho -\sigma -1}. \end{aligned}$$

### **Lemma 2**

(Kilbas and Sebastian 2008) *Let*$\lambda ,\sigma ,\vartheta \in {\mathbb {C}}$*be*$\ni \mathfrak {R}{(\lambda )}>0,\mathfrak {R}{(\rho )}<1+\min [ \mathfrak {R}{(\sigma ),\mathfrak {R}(\vartheta )}].$*Then*

19 $$\begin{aligned} \left( I_{-}^{\lambda ,\sigma ,\vartheta }t^{\rho -1}\right) (x)=\dfrac{\Gamma (\sigma -\rho +1)\Gamma (\vartheta -\rho +1)}{\Gamma (1-\rho )\Gamma (\lambda +\sigma +\vartheta -\rho +1)}x^{\rho -\sigma -1}. \end{aligned}$$

The main results are given in the following theorem.

### **Theorem 1**

*Let*$$a\in \mathbb {N}, \lambda ,\sigma ,\vartheta ,\rho ,l,b,c\in {\mathbb {C}}$$, $$\alpha >0$$*and*$$\mu$$*is an any arbitrary parameter be such that*$$\frac{l}{\xi }+\frac{b}{2}\ne -1,-2,-3,...,$$$$\mathfrak {R}{(\lambda )}>0, \mathfrak {R}{(\rho +l+1)}>\max [0,\mathfrak {R}{(\sigma -\vartheta )}]$$. *Then*

20 $$\begin{aligned}&\left( I_{0_{+}}^{\lambda ,\sigma ,\vartheta }t^{\rho -1}~_{a}{\mathcal {W}} _{l,b,c,\xi }^{\alpha ,\mu }(t)\right) (x) \nonumber \\&\quad =\dfrac{x^{l+\rho -\sigma }}{2^{l+1}} \nonumber \\&\quad \quad {\small \times {}_{3}\Psi _{4}\left[ \begin{array}{lllll} &{} (l+\rho +1,2), &{} (l+1+\rho +\vartheta -\sigma ,2), &{} (1,1) &{} \\ (\frac{l}{\xi }+\frac{b+2}{2},a), &{} (l+1+\rho -\sigma ,2), &{} (l+1+\rho +\sigma +\vartheta ,2), &{} (\mu ,\alpha ) &{} \end{array} \bigg |-\frac{cx^{2}}{4}\right] .} \end{aligned}$$

### Proof

Notice that the condition given in Eq. (17) holds for ${}_{3}\Psi _{4}$ given in (20) and then interchanging the integration and summation, (11) and (12) together imply

$$\begin{aligned} \left( I_{0_{+}}^{\lambda ,\sigma ,\vartheta }t^{\rho -1}~_{a}{\mathcal {W}} _{l,b,c,\xi }^{\alpha ,\mu }(t)\right) (x)=\sum _{k=0}^{\infty }\frac{\left( -c\right) ^{k}\left( 2\right) ^{-\left( l+2k+1\right) }}{\Gamma \left( \alpha k+\mu \right) \Gamma \left( ak+\frac{l}{\xi }+\frac{b+2}{2}\right) } \left( I_{0_{+}}^{\lambda ,\sigma ,\vartheta }t^{l+2k+\rho }\right) (x). \end{aligned}$$

For any $k=0,1,2,\ldots$, clearly $\mathfrak {R}{(l+2k+\rho +1)}\ge \mathfrak {R}{(\rho +l+1)}>\max [0,\mathfrak {R}{(\sigma -\vartheta )}]$ and hence by Lemma 1,

21 $$\begin{aligned}&\left( I_{0_{+}}^{\lambda ,\sigma ,\vartheta }t^{\rho -1}~_{a}{\mathcal {W}} _{l,b,c,\xi }^{\alpha ,\mu }(t)\right) (x) \nonumber \\& =\frac{x^{l+\rho -\sigma }}{2^{l+1}} \\&\quad \times \sum _{k=0}^{\infty }\frac{\Gamma (l+1+\rho +2k)\Gamma (l+1+\rho +\vartheta -\sigma +2k)\left( \frac{-cx^{2}}{4}\right) ^{k}}{\Gamma \left( \alpha k+\mu \right) \Gamma \left( ak+\frac{l}{\xi }+\frac{b+2}{2}\right) \Gamma (l+1+\rho -\sigma +2k)\Gamma (l+1+\rho +\lambda +\vartheta +2k)} \end{aligned}$$

In view of definition of Fox–Wright function (16) we obtain the desired result. $\square$

If we set $\alpha =a=1,\mu =\frac{3}{2}~$and $\xi =1$ in Theorem 1 then we obtain the theorem 1 of Nisar et al. (2016a) as follows:

### **Corollary 1**

*Let*$\lambda ,\sigma ,l,b,c\in {\mathbb {C}}$*be*$\ni \left( l+b/2\right) \ne -1,-2,-3\ldots$, $\mathfrak {R}(\lambda )>0$, $\mathfrak {R}(\rho +l+1)>0$. *Then*

$$\begin{aligned}&\left( I_{0_{+}}^{\lambda ,\sigma ,\vartheta }t^{\rho -1}{\mathcal {H}} _{l,b,c}(t)\right) (x) \\&\quad =\dfrac{x^{l+1+\rho -\sigma }}{2^{l+1}} \\&\quad \quad {\small \times {}_{3}\Psi _{4}\left[ \begin{array}{lllll} &{} (l+1+\rho ,2), &{} (l+1+\rho +\vartheta -\sigma ,2), &{} (1,1) &{} \\ (l+1+\frac{b}{2},1), &{} (l+1+\rho -\sigma ,2), &{} (l+1+\rho +\lambda +\vartheta ,2), &{} (\frac{3}{2},1) &{} \end{array} \bigg |-\frac{cx^{2}}{4}\right] .} \end{aligned}$$

*where*${\mathcal {H}}_{l,b,c}(t)$*is given in* (10)

### **Theorem 2**

*Let*$a\in \mathbb {N}$,$\lambda ,\sigma ,\vartheta ,\rho ,l,b,c\in {\mathbb {C}}, \alpha >0$*and*$\mu$*is an any arbitrary parameter be such that*$\left( \frac{l}{\xi }+\frac{b}{2}\right) \ne -1,-2,-3\ldots$, $\mathfrak {R}{(\lambda )}>0,$*and*$\mathfrak {R}(\rho -l)<2+\min [\mathfrak {R}(\rho ),\mathfrak {R}{(\vartheta )}]$. *Then*

22 $$\begin{aligned}&\left( I_{-}^{\lambda ,\sigma ,\vartheta }t^{\rho -1}~_{a}{\mathcal {W}} _{l,b,c,\xi }^{\alpha ,\mu }\left( \frac{1}{t}\right) \right) (x) \\&=\dfrac{x^{\rho -l-\sigma -2}}{2^{l+1}} \nonumber \\&\quad \times {}_{3}\Psi _{4}\left[ \begin{array}{rr} (l+2+\rho -\sigma ,2),(l+2+\vartheta -\rho ,2),(1,1) & \\ (\frac{l}{\xi }+\frac{b+2}{2},a),(l+2-\rho ,2),(l+2+\lambda +\sigma +\vartheta -\rho ,2),(\mu ,\alpha ) &{} \end{array} \bigg |-\frac{c}{4x^{2}}\right] . \end{aligned}$$

### Proof

The Fox–Wright function ${}_{3}\Psi _{4}$ given in (22) is well-defined as it satisfy inequality (17) and changing the order of integration and summation, (13) and (16) together imply

$$\begin{aligned} \left( I_{-}^{\lambda ,\sigma ,\vartheta }t^{\rho -1}~_{a}{\mathcal {W}}_{l,b,c,\xi }^{\alpha ,\mu }\left( \frac{1}{t}\right) \right) (x)=\sum _{k=0}^{\infty } \frac{\left( -c\right) ^{k}\left( 2\right) ^{-\left( l+2k+1\right) }}{\Gamma \left( \alpha k+\mu \right) \Gamma \left( ak+\frac{l}{\xi }+\frac{b+2}{2} \right) }\left( I_{-}^{\lambda ,\sigma ,\vartheta }t^{\rho -l-2-2k}\right) (x) \end{aligned}$$

Now using Lemma 2 and the under the conditions mentioned in Theorem 2, we have

23 $$\begin{aligned}&\left( I_{-}^{\lambda ,\sigma ,\vartheta }t^{\rho -1}~_{a}{\mathcal {W}} _{l,b,c,\xi }^{\alpha ,\mu }\left( \frac{1}{t}\right) \right) (x) \nonumber \\&\quad =\dfrac{x^{\rho -l-\sigma -2}}{2^{l+1}} \nonumber \\&\quad \times \sum _{k=0}^{\infty }\dfrac{\Gamma (\sigma -\rho +l+2+2k)\Gamma (\vartheta -\rho +l+2+2k)}{\Gamma (l+2-\rho +2k)\Gamma (\lambda +\sigma +\vartheta -\rho +l+2+2k)\Gamma \left( \alpha k+\mu \right) \Gamma \left( ak+\frac{l}{ \xi }+\frac{b+2}{2}\right) }\left( -\dfrac{c}{4x^{2}}\right) ^{k}. \end{aligned}$$

Now (22) can be deduced from (23) by using (17), hence the proof. $\square$

If we take $\alpha =a=1,\mu =\frac{3}{2}$ and $\xi =1$ in Theorem 2 then we obtain the theorem 2 of Nisar et al. (2016a) as:

### **Corollary 2**

*Let*$\lambda ,\sigma ,l,b,c\in {\mathbb {C}}$*be*$\ni \left( l+b/2\right) \ne -1,-2,-3\ldots$, $\mathfrak {R}{(\lambda )}>0,$*and*$\mathfrak {R}(\rho -l)<2+\min [\mathfrak {R}(\sigma ),\mathfrak {R}{(\vartheta )}]$. *Then*

$$\begin{aligned}&\left( I_{-}^{\lambda ,\sigma ,\vartheta }t^{\rho -1}{\mathcal {H}}_{l,b,c}\left( \frac{1}{t}\right) \right) (x) \\& =\dfrac{x^{\rho -l-\sigma -2}}{2^{l+1}} \\&\quad \times {}_{3}\Psi _{4}\left[ \begin{array}{rr} (l+2+\sigma -\rho ,2),(l+2+\vartheta -\rho ,2),(1,1) &{} \\ (l+\frac{b+1}{2},1),(l+2-\rho ,2),(l+2+\lambda +\sigma +\vartheta -\rho ,2),( \frac{3}{2},1) &{} \end{array} \bigg |-\frac{c}{4x^{2}}\right] . \end{aligned}$$

*where*${\mathcal {H}}_{l,b,c}(t)$*is given in* (10)

## Application

In this section, we infer the solution of fractional kinetic equation including generalized GTSF as an application. For this investigation, we need the following definitions:

The Swedish mathematician Mittag-Leffler introduced the so called Mittag-Leffler function $E_{\alpha }\left( z\right)$ (see Mittag-Leffler 1905):

24 $$\begin{aligned} E_{\alpha }\left( z\right) =\sum _{n=0}^{\infty }\frac{z^{n}}{\Gamma \left( \alpha n+1\right) }\quad \left( z,\alpha \in {\mathbb {C}};|z|<0,{\mathcal {R}}\left( \alpha \right) >0\right) . \end{aligned}$$

and $E_{\mu ,\eta }\left( z\right)$ defined by Wiman (1905) as

25 $$\begin{aligned} E_{\mu ,\eta }\left( z\right) =\sum _{n=0}^{\infty }\frac{z^{n}}{\Gamma \left( \mu n+\eta \right) }, \quad \left( \mu ,\eta \in {\mathbb {C}};{\mathcal {R}} \left( \mu \right)>0,{\mathcal {R}}\left( \eta \right) >0\right) . \end{aligned}$$

The familiar Riemann-Liouville fractional integral operator (see, e.g., Miller and Ross 1993; Kilbas et al. 2006) defined by

26 $$\begin{aligned} _{0}D_{t}^{-\upsilon }f(t) =\frac{1}{\Gamma \left( \upsilon \right) }\int \limits _{0}^{t}\left( t-s\right) ^{\upsilon -1}f\left( s\right) ds, \quad {\mathcal {R}}\left( \upsilon \right) >0 \end{aligned}$$

and the Laplace transform of Riemann-Liouville fractional integral operator ( Erdélyi et al. 1954; Srivastava and Saxena 2001) is

27 $$\begin{aligned} L\left\{ _{0}D_{t}^{-\upsilon }f(t) ;p\right\} =p^{-\upsilon }F\left( p\right) \end{aligned}$$

where $F\left( p\right)$ is the Laplace transform of *f*(*t*) is given by

28 $$\begin{aligned} F\left( p\right) &= {\mathcal {L}}\left\{ f(t) :p\right\} =\int _{0}^{\infty }e^{-pt}f(t) dt \\&=\lim _{\tau \rightarrow \infty }\int _{0}^{\tau }e^{-pt}f(t) dt \end{aligned}$$

whenever the limit exist (as a finite number).

### Kinetic equations

The standard kinetic equation is of the form,

29 $$\begin{aligned} \frac{dN_{i}}{dt}=-c_{i}N_{i}(t) \end{aligned}$$

with $N_{i}\left( t=0\right) =N_{0}$, which is the number of density of species *i* at time $t=0$ and $c_{i}>0$. The integration of (29) gives an alternate form as follows:

30 $$\begin{aligned} N(t) -N_{0}=-c.~_{0}D_{t}^{-1}N(t) \end{aligned}$$

where $_{0}D_{t}^{-1}$ is the special case of the Riemann-Liouville integral operator and c is a constant. The fractional generalization of (30) is given by Haubold and Mathai (2000) as:

31 $$\begin{aligned} N(t) -N_{0}=-c_{0}^{\upsilon }D_{t}^{-\upsilon }N(t) \end{aligned}$$

where $_{0}D_{t}^{-\upsilon }$ defined in (26).

Recently, Saxena and Kalla (2008) considered the following equation

32 $$\begin{aligned} N(t) -N_{0}f(t) =-c^{v}.~D_{t}^{-v}N(t) ,\quad Re\left( v\right)>0,c>0 \end{aligned}$$

and obtained the solution as:

33 $$\begin{aligned} N(t) =N_{0}\sum _{k=0}^{\infty }\left( -1\right) ^{k}\frac{c^{kv} }{\Gamma \left( kv\right) }t^{kv-1}*f(t) \end{aligned}$$

where

$$\begin{aligned} t^{kv-1}*f(t) =\int _{0}^{t}\left( t-u\right) ^{kv-1}f\left( u\right) du. \end{aligned}$$

For more details about the solution of kinetic equations interesting readers can refer (Saxena and Kalla 2008; Nisar and Atangana 2016).

### Solution of fractional kinetic equation involving (11)

In this section, we will discuss about the solution fractional kinetic equation involving newly defined function generalized GTSF to show the potential of newly defined function in application level.

Given the equation

34 $$\begin{aligned} N(t) -N_{0}~_{a}{\mathcal {W}}_{l,b,c,\xi }^{\alpha ,\mu }(t) :=-e^{\upsilon }_{0}D_{t}^{-\upsilon }N(t) , \end{aligned}$$

where $$e,t,v\in R^{+},a,b,c,l\in {\mathbb {C}}$$ and $${\mathcal {R}}\left( l\right) >-1.$$

Taking the Laplace transform of (34) and using (11) and (27), gives

35 $$\begin{aligned} {\mathcal {N}}\left( p\right) &= N_{0}\left( \int _{0}^{\infty }e^{-pt}\sum _{k=0}^{\infty }\frac{\left( -c\right) ^{k}}{\Gamma \left( \alpha k+\mu \right) \Gamma \left( ak+\frac{l}{\xi }+\frac{b+2}{2}\right) } \left( \frac{t}{2}\right) ^{2k+l+1}\right) dt \\&\quad -e^{\upsilon }p^{-\upsilon }{\mathcal {N}}\left( p\right) \end{aligned}$$

where $${\mathcal {N}}\left( p\right) =L\left\{ N(t) ;p\right\}$$

Integrate the integral in (35) term by term which guaranteed under the given restrictions and using (5), we get: for $Re\left( p\right) >0$

$$\begin{aligned} \left( 1+\left( \frac{e}{p}\right) ^{v}\right) {\mathcal {N}}\left( p\right) =N_{0}\sum _{k=0}^{\infty }\frac{\left( -c\right) ^{k}2^{-\left( 2k+l+1\right) }}{\Gamma \left( \alpha k+\mu \right) \Gamma \left( ak+\frac{l }{\xi }+\frac{b+2}{2}\right) }\frac{\Gamma \left( 2k+l+2\right) }{p^{2k+l+2}} \end{aligned}$$

Taking the geometric series expansion of $\left( 1+\left( \frac{e}{p}\right) ^{v}\right) ^{-1},$ we have: for $e<\left| p\right|$

36 $$\begin{aligned} {\mathcal {N}}\left( p\right)& = N_{0}\sum \limits _{k=0}^{\infty }\frac{\left( -c\right) ^{k}\left( 2\right) ^{-\left( 2k+l+1\right) }\Gamma \left( 2k+l+2\right) }{\Gamma \left( \alpha k+\mu \right) \Gamma \left( ak+\frac{l}{ \xi }+\frac{b+2}{2}\right) p^{\left( 2k+l+2\right) }} \\&\quad \times \sum \limits _{r=0}^{\infty }\left( -1\right) ^{r}\left( \frac{e}{p} \right) ^{vr} \end{aligned}$$

Applying the inverse Laplace transform and using the following known formula:

37 $$\begin{aligned} L^{-1}[p^{-\upsilon }]=\frac{t^{\upsilon -1}}{\Gamma \left( \upsilon \right) }, \quad{\mathcal {R}}\left( \upsilon \right) >0 \end{aligned}$$

we have

$$\begin{aligned} N(t)& = L^{-1}\left\{ {\mathcal {N}}\left( p\right) \right\} \\&= N_{0}\sum \limits _{k=0}^{\infty }\frac{\left( -c\right) ^{k}\Gamma \left( 2k+l+2\right) }{\Gamma \left( \alpha k+\mu \right) \Gamma \left( ak+\frac{l}{ \xi }+\frac{b+2}{2}\right) }\left( \frac{t}{2}\right) ^{2k+l+1} \\& \quad \times \left\{ \sum \limits _{r=0}^{\infty }\frac{\left( -1\right) ^{r}\left( et\right) ^{\upsilon r}}{\Gamma \left( \upsilon r+l+2k+2\right) } \right\} \end{aligned}$$

In view of Eq. (25), we get,

38 $$\begin{aligned} N(t) =N_{0}\sum _{k=0}^{\infty }\frac{\left( -c\right) ^{k}\Gamma \left( 2k+l+2\right) }{\Gamma \left( \alpha k+\mu \right) \Gamma \left( ak+ \frac{l}{\xi }+\frac{b+2}{2}\right) }\left( \frac{t}{2}\right) ^{2k+l+1}E_{v,2k+l+2}\left( -e^{\upsilon }t^{\upsilon }\right) . \end{aligned}$$

The following results are more general than (38) and they can derive parallel as above, so the details are omitted.

**Fig. 1:**
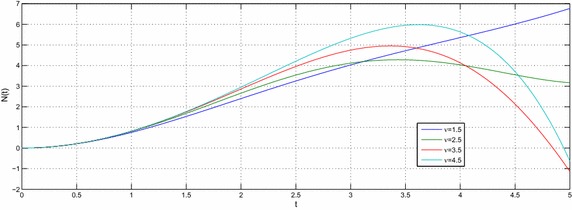
Solution (38) for $a=1$, $N_{0}=1, \alpha =\mu =\xi =1$ and $b=c=l=e=1$

**Fig. 2:**
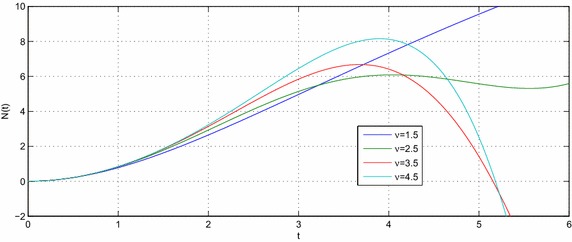
Solution (38) for $a=2$, $N_{0}=1,\,\alpha =\mu =\xi =1$ and $b=c=l=e=1$

**Fig. 3:**
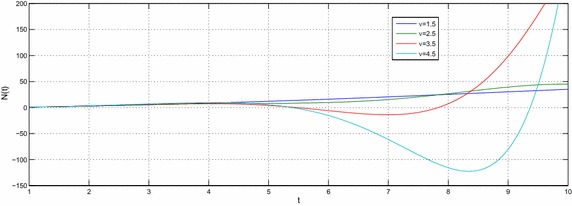
Solution (38) for $a=3$, $N_{0}=1, \alpha =\mu =\xi =1$ and $b=c=l=e=1$

**Fig. 4:**
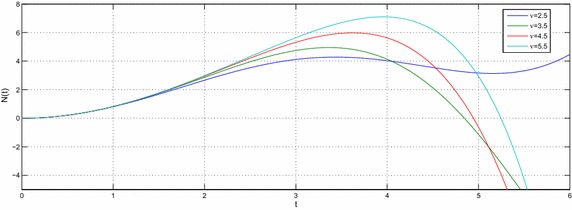
Solution (40) for $a=1$, $N_{0}=1,\,\alpha =\mu =\xi =1$ and $b=c=l=e=1$

**Fig. 5:**
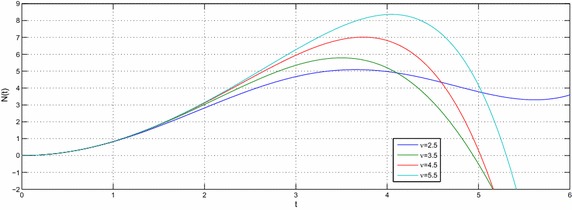
Solution (38) for $a=1.5$, $N_{0}=1$, $\alpha =\mu =\xi =1$ and $b=c=l=e=1$

**Fig. 6:**
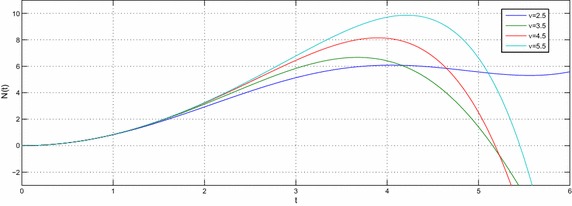
Solution (40) for $a=2$, $N_{0}=1$, $\alpha =\mu =\xi =1$ and $b=c=l=e=1$

**Fig. 7:**
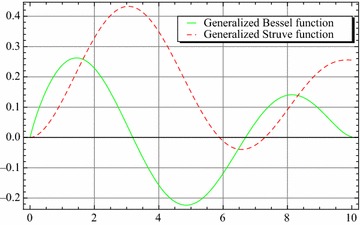
Comparison between the Solution (38) for $\nu =\frac{1}{2}, a=1$, $N_{0}=1$, $\alpha =\mu =\xi =1$ and $b=c=l=e=1$ and (18) of Kumar et al. (2015)

Let $e,t,v\in R^{+},a,b,c,l\in {\mathbb {C}}$ with ${\mathcal {R}}(l)>-1$ then the equation

39 $$\begin{aligned} N(t) -N_{0}~_{a}{\mathcal {W}}_{p,b,c,\xi }^{\alpha ,\mu }\left( e^{\upsilon }t^{\upsilon }\right) =-e^{\upsilon }{}_{0}D_{t}^{-\upsilon }N(t) \end{aligned}$$

have the following solution

40 $$\begin{aligned} N(t) =N_{0}\sum \limits _{k=0}^{\infty }\frac{\left( -c\right) ^{k}\Gamma \left( 2k\nu +\upsilon l+\nu +1\right) }{\Gamma \left( \alpha k+\mu \right) \Gamma \left( ak+\frac{l}{\xi }+\frac{b+2}{2}\right) }\left( \frac{t^{^{\upsilon }}e^{\upsilon }}{2}\right) ^{2k+l+1}E_{v,\left( 2k+l+1\right) \upsilon +1}\left( -e^{\upsilon }t^{\upsilon }\right) \end{aligned}$$

and the solution of the equation

41 $$\begin{aligned} N(t) -N_{0}~_{a}{\mathcal {W}}_{p,b,c,\xi }^{\alpha ,\mu }\left( e^{\upsilon }t^{\upsilon }\right) =-{\mathfrak {a}}^{\upsilon }{}_{0}D_{t}^{-\upsilon }N(t) \end{aligned}$$

is

42 $$\begin{aligned} N(t) & = N_{0}\sum \limits _{k=0}^{\infty }\frac{\left( -c\right) ^{k}\Gamma \left( 2k\upsilon +\upsilon l+\upsilon +1\right) }{\Gamma \left( \alpha k+\mu \right) \Gamma \left( ak+\frac{l}{\xi }+\frac{b+2}{2}\right) } \left( \frac{e^{\upsilon }}{2}\right) ^{2k+l+1} \\& \quad \times t^{\upsilon \left( 2k+l+1\right) }E_{v,\left( 2k+l+1\right) \upsilon +1}\left( -{\mathfrak {a}}^{\upsilon }t^{\upsilon }\right) \end{aligned}$$

where ${\mathfrak {a}}\ne e$. The Figs. 1, 2, 3, 4, 5 and 6 are presented to show the behavior of the solution *N*(*t*) for different values of *a* and $\nu$. The comparison between solutions of GFKE involving generalized Bessel function (solid green line) and generalized Galué type generalization of Struve function (dashed red line) are shown in Fig. 7.

## Conclusion

In this paper, we investigated the integral transforms of Galué type generalization of Struve function and the results expressed in terms of Fox–Wright function. By substituting the appropriate value for the parameters, we obtained some results existing in the literature as corollaries. The results derived in section "Application" of this paper are general in character and likely to find certain applications in the theory of fractional calculus and special functions. The solutions of certain general families of fractional kinetic equations involving generalized GTSF presented in section "Conclusion". The main results given in section "Solution of fractional kinetic equation involving (11)" are general enough to be specialized to yield many new and known solutions of the corresponding generalized fractional kinetic equations. For instance, if we put $a=\alpha =\xi =1$ and $\mu =\frac{3}{2}$ in (34), (39) and (41), then we get the Eqs. (15), (19) and (24) of Nisar et al. (2016b).
